# Exploring Meat Attachment and Consumption Patterns in Primary Care Patients: Insights From the Four-Item Meat Attachment Questionnaire

**DOI:** 10.7759/cureus.103936

**Published:** 2026-02-19

**Authors:** Paul Sebo, Bruno Delaunay, Benoit Tudrej, Mohamed Amir Moussa, Hubert Maisonneuve

**Affiliations:** 1 University Institute for Primary Care, University of Geneva, Geneva, CHE; 2 University College of General Medicine, University Claude Bernard Lyon 1, Lyon, FRA

**Keywords:** maq, meat attachment, meat attitudes, meat consumption, primary care, switzerland

## Abstract

Background: The 16-item Meat Attachment Questionnaire measures emotional and cognitive attachment to meat across four dimensions: hedonism, affinity, entitlement, and dependence. A shorter version, the four-item Meat Attachment Questionnaire (MAQ-4), with scores ranging from 4 to 20, has recently been validated in French (MAQf-4). This study is a planned secondary analysis of an existing dataset and examines the association between meat attachment, as measured by the MAQf-4, and meat consumption patterns among primary care patients in the Geneva area.

Methods: This cross-sectional study surveyed 336 primary care patients (204, 61.3%, women; participation rate = 79.1%) from the Geneva area, collecting data on demographics, meat consumption frequencies (poultry, beef, veal, and pork), and MAQf-4 scores. Differences in MAQf-4 scores by demographic characteristics were analyzed using independent t-tests, analysis of variance (ANOVA), and multivariable linear regressions. Additionally, ANOVA and multivariable linear regressions assessed the relationship between MAQf-4 scores and meat consumption patterns.

Results: The average MAQf-4 score was 13.6 (standard deviation (SD) 3.6), with men scoring higher than women (14.6 vs. 13.0, adjusted difference: 1.9 (95% confidence interval (CI) 1.2-2.6), adjusted *P*-value <0.001). Poultry was the most frequently consumed meat, with 124 (39%) participants eating it more than once a week. Higher MAQf-4 scores were significantly associated with greater meat consumption across all categories; for instance, those consuming poultry more than once a week had a mean score of 14.6 compared to 9.9 for non-consumers (adjusted difference: 4.2 (95% CI 3.0-5.5), adjusted *P*-value < 0.001).

Conclusions: The MAQf-4 is strongly associated with meat consumption patterns. Its brevity makes it a potentially useful tool for research and clinical settings to assess meat attachment.

## Introduction

Meat is a staple in many diets around the world, providing essential nutrients such as protein, iron, and vitamin B12 [1,2]. However, excessive meat consumption, particularly of red and processed meats, is associated with numerous health issues, including cardiovascular disease [3,4], type 2 diabetes [4], and certain cancers [5]. The environmental impact of meat production, including greenhouse gas emissions and resource-intensive farming, has also led to calls for reduced meat consumption [6]. Despite these concerns, reducing meat intake remains challenging due to the cultural, emotional, and psychological significance attached to meat [7,8].

The 16-item Meat Attachment Questionnaire (MAQ-16) was developed to measure the psychological attachment to meat across four dimensions: hedonism (enjoyment of eating meat), affinity (emotional connection to meat), entitlement (perceived right to eat meat), and dependence (difficulty imagining life without meat) [8]. It has been translated and validated in French [9]. While comprehensive, the MAQ-16 can be burdensome in contexts where time and participant fatigue are concerns. Therefore, a shorter version, the four-item Meat Attachment Questionnaire (MAQ-4), was created, retaining one item from each dimension of the MAQ-16. Validated in French (MAQf-4), this version offers a more practical option for both research and clinical use [10].

Although the French version of the 16-item MAQ (MAQf-16) has been shown to correlate with actual meat consumption [11], the present study constitutes a planned secondary analysis of the same cohort, designed to assess the association between MAQf-4 scores and meat consumption patterns in a primary care population. By clarifying this relationship, the study may inform future efforts to support more tailored dietary counselling in clinical settings.

## Materials and methods

Study design and setting

This cross-sectional study was conducted from January to May 2024 among patients attending primary care practices in the Geneva area, Switzerland. The primary objective was to assess the association between meat attachment, as measured by the MAQf-4, and meat consumption patterns. This paper reports a planned secondary analysis of a dataset previously collected in primary care to study meat attachment using the MAQf-16 [11]. The same cohort was intentionally used to ensure that the MAQf-4 was evaluated under identical conditions to those of the previously published MAQf-16 study.

Participants

**Figure 1:**
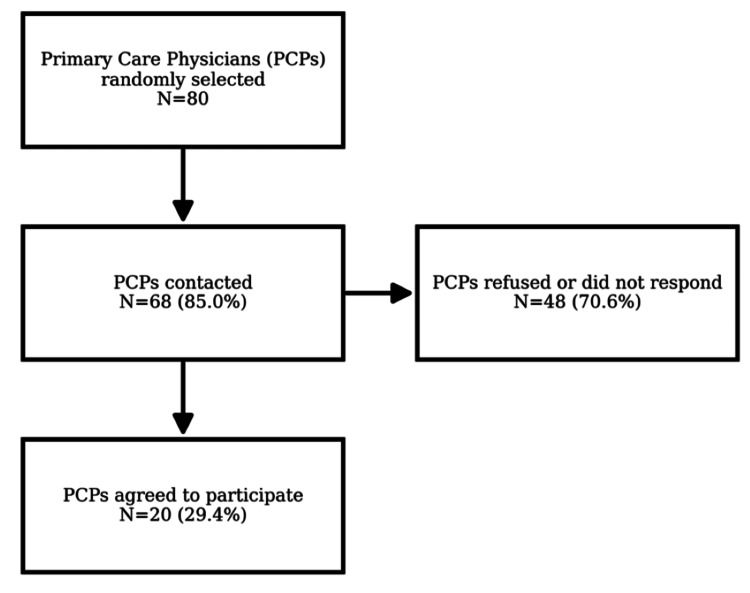
Flowchart of primary care physician recruitment.

**Figure 2:**
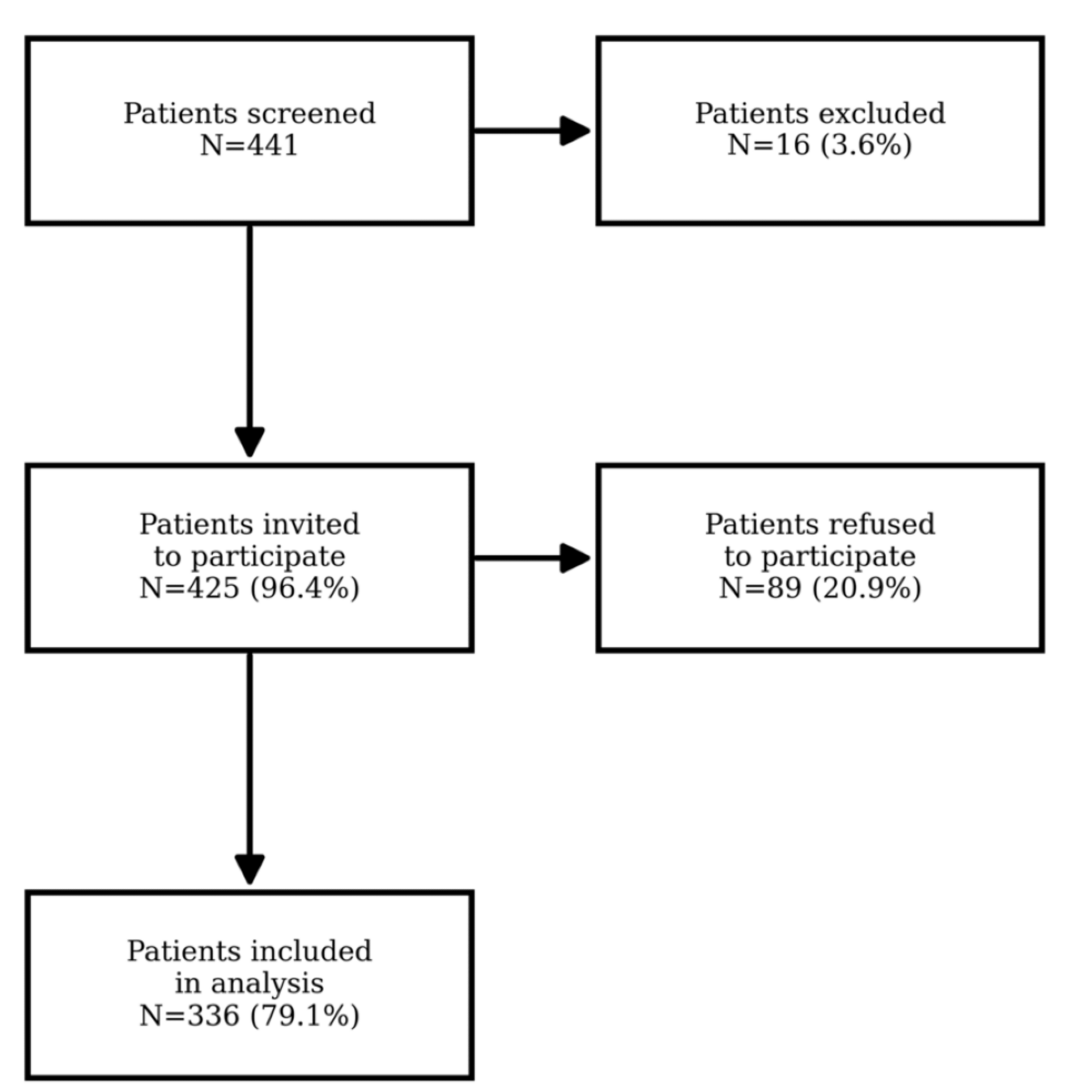
Flowchart of patient recruitment.

Figures 1-2 illustrate the recruitment process. Figure 1 presents the selection of participating primary care physicians (PCPs), who were randomly selected from the regional registry of practicing PCPs in the Geneva area, and Figure 2 details patient recruitment. Among the 20 physicians who took part in the study, 425 patients attending their practices were invited, of whom 336 (79%) consented to participate. Patients were recruited consecutively during routine consultations to minimize selection bias. Eligible patients were adults (≥18 years) with sufficient French proficiency to complete the questionnaire.

Data collection

Data were collected through a self-administered questionnaire distributed in the waiting rooms of participating practices. The questionnaire was identical to that used in the previously published MAQf-16 paper [11] and included the full MAQf-16; MAQf-4 scores were subsequently derived from four selected MAQf-16 items for the purpose of this planned secondary analysis.

The questionnaire collected demographic information (gender, age, place of residence, and occupation), self-reported frequencies of meat consumption (poultry, beef, veal, and pork) using the meat and fish section of the Food Frequency Questionnaire developed for the EPIC study [12], and MAQf-16 responses from which MAQf-4 scores were computed.

Meat Attachment Questionnaire (MAQf-4)

The MAQf-4 consists of four items derived from the original 16-item questionnaire, which assesses attachment to meat consumption across four key dimensions: hedonism, affinity, entitlement, and dependence [10].

Hedonism is captured by MAQf-16 item 5, “I love meals with meat” (« J’adore les repas avec de la viande »). Dependence is reflected by item 12, “If I was forced to stop eating meat I would feel sad” (« Si on m’obligeait à cesser de manger de la viande, je serais triste »). Affinity is represented by item 14, “By eating meat I’m reminded of the death and suffering of animals” (« En mangeant de la viande, je pense à la mort et à la souffrance des animaux »). Entitlement is measured using item 15a, “Eating meat is a natural practice” (« Manger de la viande est une pratique naturelle »).

Each item is rated on a 5-point Likert scale with the following response options: 1 = strongly disagree (pas du tout d’accord), 2 = disagree (plutôt pas d’accord), 3 = neither agree nor disagree (ni d’accord ni pas d’accord), 4 = agree (plutôt d’accord), and 5 = strongly agree (tout à fait d’accord). Item 14 is reverse-coded. MAQf-4 total scores range from 4 to 20, with higher scores indicating stronger attachment to meat; mean scores were computed as the average of the four items. In this study, we analyzed both mean and total scores.

Ethical considerations

This study was conducted in compliance with the Declaration of Helsinki. Informed consent was obtained from all participants before their inclusion in the study. Ethical approval was granted by the Research Ethics Committee of the University College of General Practice at Claude Bernard University (Project ID: IRB 2023-01-03-01), with an additional waiver provided by the Cantonal Research Ethics Commission of Geneva (Project ID: 2023-01941). The secondary analyses reported in this manuscript were conducted under the same approved ethical protocol.

Statistical analysis

Descriptive statistics were used to summarize participant characteristics, meat consumption patterns, and MAQf-4 mean and total scores. The MAQf-4 scores were found to follow a normal distribution, as confirmed by graphical methods and the Shapiro-Francia test. Homogeneity of variances was verified using Bartlett’s test, and we also examined the normality and homoscedasticity of the residuals.

Differences in MAQf-4 scores based on demographic characteristics were analyzed using independent t-tests, ANOVA, and multivariable linear regressions. Additionally, analysis of variance (ANOVA) and multivariable linear regressions were conducted to assess the relationship between MAQf-4 scores and meat consumption patterns. In the multivariable analyses, adjustments were made for age, gender, residence, and occupation. Regression models accounted for intra-cluster correlation at the practice level using cluster-robust standard errors. Statistical significance was defined as *P*-values < 0.05.

Missing data were handled using a complete-case approach. Analyses were conducted using all available observations for the variables included in each model. The proportion of missing data was low, and no imputation procedures were performed. All analyses were performed using STATA 17.0 (StataCorp, College Station, TX).

## Results

Participants' characteristics

As shown in Table 1, the sample included 204 women (61%), with a median participant age of 53 years (range: 18-94 years). Most participants were employed (*n* = 109, 33%), while 89 (27%) were retired and 46 (14%) were unemployed. Although 336 patients were included in the study, some participants did not provide information for all sociodemographic variables. Consequently, the number of observations varies slightly across characteristics in Table 1, and descriptive statistics were calculated based on the available data for each variable.

**Table 1:** Patients’ characteristics.

Characteristic	*n* (%)	Median (IQR)	Min-max
Gender (*n* = 333)			
Female	204 (61.3)		
Male	127 (38.1)		
Other	2 (0.6)		
Age (years) (*n* = 330)		53 (28)	18-94
<40	88 (26.7)		
40-59	119 (36.0)		
≥60	123 (37.3)		
Residence (*n* = 325)			
Geneva	277 (85.2)		
Vaud	22 (6.8)		
France	18 (5.5)		
Other	8 (2.5)		
Occupation (*n* = 329)			
Employee	109 (33.1)		
Retired	89 (27.0)		
Unemployed	46 (14.0)		
Managerial and professional occupations	43 (13.1)		
Intermediate-level profession	24 (7.3)		
Other	18 (5.5)		

Meat attachment scores

Meat attachment scores are presented in Table 2. As in Table 1, the number of observations in Table 2 varies due to missing data; all analyses were therefore based on the available data for each variable. The overall mean score was 3.4 (SD = 0.9, range: 1-5), and the overall total score was 13.6 (SD = 3.6, range: 4-20). Men reported significantly higher scores (mean = 3.6, total = 14.6) compared to women (mean = 3.2, total = 13.0), with adjusted *P*-values < 0.001. No significant differences in scores were found across age groups (adjusted *P*-values = 0.08).

**Table 2:** MAQf-4 mean and total scores, and levels of poultry, beef, veal, and pork consumption, overall and stratified by gender and age group. Mean (SD) for MAQf-4 and *n* (%) for meat consumption. ^a^t-tests for MAQ mean and total scores, and chi-square tests for meat consumption. ^b^Multivariable linear regressions for MAQ mean and total scores, multivariable logistic regressions for meat consumption (two categories: <1 time/week and ≥1 time/week); models adjusted for age group, gender, residence, occupation, and intra-cluster correlation within practices. ^c^ANOVA for MAQ mean and total scores, chi-square tests for meat consumption. ^d^Multivariable linear regressions for MAQ mean and total scores, multivariable logistic regressions for meat consumption (two categories: <1 time/week and ≥1 time/week); models adjusted for age group, gender, residence, occupation, and intra-cluster correlation within practices. ANOVA, analysis of variance; MAQf, Meat Attachment Questionnaire validated in French

Variable	Overall	Female	Male	Unadjusted *P*-value^a^	Adjusted *P*-value^b^	<40 years	40-59 years	≥60 years	Unadjusted *P*-value^c^	Adjusted *P*-value^d^
MAQf-4 mean score (*n *= 321)	3.4 (0.9)	3.2 (0.1)	3.6 (0.1)	<0.001	<0.001	3.6 (0.8)	3.4 (0.8)	3.2 (0.9)	0.01	0.08
MAQf-4 total score (*n *= 321)	13.6 (3.6)	13.0 (0.3)	14.6 (0.3)	<0.001	<0.001	14.5 (3.3)	13.4 (3.6)	13.0 (3.5)	0.01	0.08
Poultry consumption (*n *= 318)				0.07	0.02				<0.001	0.07
Never	29 (9.1)	19 (9.7)	9 (7.7)			3 (3.6)	9 (7.9)	17 (14.3)		
<1 time/week	77 (24.2)	53 (26.9)	23 (19.5)			17 (20.2)	22 (19.5)	38 (31.9)		
1 time/week	88 (27.7)	45 (22.8)	43 (36.4)			20 (23.8)	29 (25.7)	37 (31.1)		
>1 time/week	124 (39.0)	80 (40.6)	43 (36.4)			44 (52.4)	53 (46.9)	27 (22.7)		
Beef consumption (*n *= 318)				0.01	0.001				0.09	0.10
Never	51 (16.0)	39 (19.8)	11 (9.3)			12 (14.3)	12 (10.6)	27 (22.7)		
<1 time/week	115 (36.2)	77 (39.1)	37 (31.4)			25 (29.8)	47 (41.6)	43 (36.1)		
1 time/week	96 (30.2)	54 (27.4)	42 (35.6)			27 (32.1)	34 (30.1)	34 (28.6)		
>1 time/week	56 (17.6)	27 (13.7)	28 (23.7)			20 (23.8)	20 (17.7)	15 (12.6)		
Veal consumption (*n *= 314)				0.03	0.03				0.42	0.39
Never	130 (41.4)	92 (47.4)	37 (31.6)			41 (49.4)	41 (37.3)	48 (40.3)		
<1 time/week	110 (35.0)	64 (33.0)	45 (38.5)			24 (28.9)	38 (34.5)	47 (39.5)		
1 time/week	37 (11.8)	18 (9.3)	19 (16.2)			7 (8.4)	16 (14.6)	13 (10.9)		
>1 time/week	37 (11.8)	20 (10.3)	16 (13.7)			11 (13.3)	15 (13.6)	11 (9.3)		
Pork consumption (*n *= 314)				0.003	0.05				0.86	0.99
Never	138 (44.0)	100 (51.5)	37 (31.6)			34 (40.5)	48 (43.6)	56 (47.4)		
<1 time/week	98 (31.2)	57 (29.4)	40 (34.2)			27 (32.1)	32 (29.1)	38 (32.2)		
1 time/week	50 (15.9)	23 (11.9)	27 (23.1)			15 (17.9)	20 (18.2)	14 (11.9)		
>1 time/week	28 (8.9)	14 (7.2)	13 (11.1)			8 (9.5)	10 (9.1)	10 (8.5)		

Table 3 shows the unadjusted and adjusted predicted differences in mean and total scores by gender and age group. The adjusted difference for men compared to women in mean and total scores was 0.5 (95% CI 0.3-0.6) and 1.9 (95% CI 1.2-2.6), respectively, with adjusted *P*-values <0.001.

**Table 3:** Predicted differences in MAQf-4 mean and total scores by gender, age group, and levels of poultry, beef, veal, and pork consumption. ^a^Univariable linear regressions; models adjusted for intra-cluster correlation within practices. ^b^Multivariable linear regressions; models adjusted for age group, gender, residence, occupation, and intra-cluster correlation within practices. MAQf, Meat Attachment Questionnaire validated in French; CI, confidence interval

Variable	Unadjusted difference in MAQf-4 mean score (95% CI)	*P*-value^a^	Adjusted difference in MAQf-4 mean score (95% CI)	*P*-value^b^	Unadjusted difference in MAQf-4 total score (95% CI)	*P*-value^a^	Adjusted difference in MAQf-4 total score (95% CI)	*P*-value^b^
Gender		<0.001		<0.001		<0.001		<0.001
Female	0		0		0		0	
Male	0.40 (0.21-0.59)		0.47 (0.30-0.64)		1.59 (0.83-2.35)		1.87 (1.18-2.57)	
Age group		0.03		0.08		0.03		0.08
<40 years	0.39 (0.11-0.67)		0.41 (0.02-0.80)		1.57 (0.45-2.69)		1.63 (0.06-3.20)	
40-59 years	0.12 (-0.17 to 0.41)		0.16 (-0.21 to 0.53)		0.49 (-0.68 to 1.65)		0.64 (-0.83 to 2.10)	
≥60 years	0		0		0		0	
Poultry consumption		<0.001		<0.001		<0.001		<0.001
Never	0		0		0		0	
<1 time/week	0.62 (0.33-0.90)		0.57 (0.31-0.82)		2.46 (1.30-3.62)		2.27 (1.25-3.29)	
1 time/week	1.09 (0.73-1.45)		0.93 (0.58-1.29)		4.36 (2.93-5.80)		3.74 (2.33-5.14)	
>1 time/week	1.16 (0.83-1.48)		1.06 (0.75-1.36)		4.62 (3.33-5.90)		4.23 (3.00-5.46)	
Beef consumption		<0.001		<0.001		<0.001		<0.001
Never	0		0		0		0	
<1 time/week	0.62 (0.29-0.95)		0.54 (0.21-0.87)		2.47 (1.16-3.78)		2.16 (0.86-3.46)	
1 time/week	0.92 (0.54-1.31)		0.81 (0.44-1.18)		3.69 (2.15-5.24)		3.24 (1.77-4.71)	
>1 time/week	1.19 (0.86-1.51)		1.03 (0.67-1.38)		4.74 (3.44-6.05)		4.10 (2.70-5.50)	
Veal consumption		<0.001		<0.001		<0.001		<0.001
Never	0		0		0		0	
<1 time/week	0.47 (0.21-0.73)		0.45 (0.21-0.68)		1.89 (0.85-2.92)		1.79 (0.85-2.74)	
1 time/week	0.45 (0.12-0.78)		0.42 (0.07-0.78)		1.80 (0.48-3.13)		1.69 (0.28-3.10)	
>1 time/week	0.85 (0.53-1.17)		0.74 (0.41-1.07)		3.40 (2.11-4.68)		2.96 (1.66-4.26)	
Pork consumption		<0.001		<0.001		<0.001		<0.001
Never	0		0		0		0	
<1 time/week	0.47 (0.31-0.62)		0.41 (0.25-0.57)		1.86 (1.24-2.49)		1.65 (1.00-2.30)	
1 time/week	0.55 (0.33-0.78)		0.48 (0.29-0.68)		2.22 (1.32-3.12)		1.93 (1.14-2.71)	
>1 time/week	0.71 (0.23-1.20)		0.64 (0.22-1.05)		2.86 (0.93-4.78)		2.54 (0.87-4.21)	

Meat consumption patterns

Meat consumption data are reported here because they are required to evaluate the concurrent validity of the MAQf-4; the same cohort and consumption dataset were used in the previously published MAQf-16 paper [11]. Poultry was the most commonly consumed meat, with 39% of participants reporting consumption more than once per week (Table 2). Beef was consumed more than once per week by 18% of participants, while veal and pork were less frequently consumed. Men reported higher consumption across all meat types compared to women, with adjusted *P*-values < 0.05 for all comparisons.

Association between MAQf-4 scores and meat consumption

Higher MAQf-4 scores were consistently associated with greater meat consumption (Table 4). For instance, individuals consuming poultry more than once a week had mean and total MAQf-4 scores of 3.6 and 14.6, respectively, compared to scores of 2.5 and 9.9 for those who never consumed poultry (adjusted *P*-values < 0.001). Similar patterns were observed for beef, veal, and pork, with higher MAQf-4 scores correlating with more frequent consumption. As these analyses are based on associations between meat consumption and MAQf-4 scores, only participants with complete data for both variables were included, resulting in slightly smaller sample sizes compared with the descriptive data.

**Table 4:** Association between MAQf-4 mean and total scores, and levels of poultry, beef, veal, and pork consumption. ^a^ANOVA. ^b^Multivariable linear regressions; models adjusted for age group, gender, residence, occupation, and intra-cluster correlation within practices. ANOVA, analysis of variance; MAQf, Meat Attachment Questionnaire validated in French

Variable	n	MAQf-4 mean score (SD)	Unadjusted *P*-value^a^	Adjusted *P*-value^b^	MAQf-4 total score (SD)	Unadjusted *P*-value^a^	Adjusted *P*-value^b^
Poultry consumption			<0.001	<0.001		<0.001	<0.001
Never	29	2.5 (0.8)			9.9 (3.3)		
<1 time/week	77	3.1 (0.9)			12.4 (3.4)		
1 time/week	88	3.6 (0.8)			14.3 (3.1)		
>1 time/week	122	3.6 (0.8)			14.6 (3.3)		
Beef consumption			<0.001	<0.001		<0.001	<0.001
Never	51	2.7 (0.9)			10.7 (3.7)		
<1 time/week	114	3.3 (0.7)			13.2 (2.8)		
1 time/week	95	3.6 (0.9)			14.4 (3.6)		
>1 time/week	56	3.9 (0.7)			15.4 (3.0)		
Veal consumption			<0.001	0.001		<0.001	0.001
Never	129	3.1 (0.9)			12.3 (3.7)		
<1 time/week	110	3.5 (0.8)			14.2 (3.1)		
1 time/week	36	3.5 (0.8)			14.1 (3.4)		
>1 time/week	37	3.9 (0.8)			15.7 (3.0)		
Pork consumption			<0.001	<0.001		<0.001	<0.001
Never	136	3.1 (0.9)			12.3 (3.6)		
<1 time/week	98	3.6 (0.8)			14.2 (3.0)		
1 time/week	50	3.6 (0.9)			14.5 (3.5)		
>1 time/week	28	3.8 (0.8)			15.2 (3.3)		

The unadjusted and adjusted predicted differences in mean and total scores by levels of poultry, beef, veal, and pork consumption are shown in Table 3. For example, the adjusted difference in mean and total scores for those consuming poultry more than once a week compared to those who never consumed poultry was 1.1 (95% CI 0.8-1.4) and 4.2 (95% CI 3.0-5.5), respectively, with adjusted *P*-values <0.001.

## Discussion

Main findings

The present paper reports a planned secondary analysis focusing on the short-form MAQf-4. This study shows that the MAQf-4 is strongly associated with meat consumption patterns among primary care patients, corroborating similar findings obtained with the MAQf-16 [11]. However, the cross-sectional nature of the study precludes any causal inference regarding the relationship between meat attachment and meat consumption. Individuals with higher attachment to meat, as measured by the MAQf-4, reported consuming more meat across all categories examined. Furthermore, men had higher MAQf-4 scores and reported greater meat consumption compared to women.

Comparison with existing literature

These findings are consistent with previous research indicating that strong emotional and cognitive attachments to meat are associated with higher consumption levels [11,13]. Studies using the 16-item MAQ have reported similar associations, underscoring the role of psychological attachment as a barrier to reducing meat intake [11,13]. The current study extends these insights to the MAQf-4, highlighting its potential utility for brief assessment in clinical settings.

The observed gender differences in meat attachment and consumption align with existing literature. Men consistently reported stronger attachments to meat and higher levels of consumption [14,15], likely reflecting cultural norms associating meat-eating with masculinity [16,17]. These findings suggest that dietary interventions may need to be tailored to address these sociocultural factors.

Clinical implications

The MAQf-4 may provide a practical, time-efficient tool for clinicians to assess patients' attachment to meat. By identifying individuals with a high attachment to meat, healthcare providers may be better positioned to offer more personalized dietary counseling that addresses the emotional and cognitive barriers to reducing meat consumption. For example, motivational interviewing techniques could be employed to explore patients' values and beliefs about meat, potentially facilitating behavior change.

Given the health and environmental benefits of reducing meat consumption [6], incorporating the MAQf-4 into primary care assessments may support broader public health efforts. Clinicians may consider using this tool to guide discussions about the health risks associated with excessive meat consumption and the benefits of plant-based diets, contributing to more personalized and effective dietary advice.

Methodological considerations and future research

The cross-sectional nature of this study limits the ability to establish causality between meat attachment and consumption patterns. Longitudinal studies are needed to explore whether changes in meat attachment, as measured by the MAQf-4, lead to subsequent changes in consumption. Additionally, research in diverse populations and settings would help validate these findings and explore cultural differences in meat attachment.

Future studies could also investigate the effectiveness of interventions aimed at reducing meat attachment and consumption, using the MAQf-4 as an outcome measure. Such research may provide valuable insights into the psychological and behavioral processes involved in dietary change, informing the design of more effective public health campaigns and clinical interventions.

Moreover, the study relied on self-reported data, which may introduce recall bias or social desirability bias, especially in dietary reporting. Future research should consider the inclusion of objective measures of meat consumption, such as food diaries [18] or biomarkers [19,20], to corroborate self-reported data and provide a more accurate picture of meat consumption behavior.

Further work is also needed to assess the sensitivity of the MAQf-4 in various demographic groups, particularly those with specific dietary preferences, such as vegetarians, vegans, or flexitarians. Understanding how the MAQf-4 performs in populations with a pre-existing reduced meat intake could reveal nuances in meat attachment across different lifestyle choices.

Finally, the impact of educational, environmental, and cultural interventions on reducing meat attachment and subsequent meat consumption should be explored. These interventions could include public health campaigns focusing on the environmental impacts of meat production or emphasizing the health risks of excessive meat intake. Studies could measure whether exposure to such interventions decreases meat attachment scores and whether this results in meaningful dietary changes.

Limitations

This study has several limitations that must be acknowledged when interpreting the findings, some of which arise from the methodological considerations outlined above. First, the cross-sectional design prevents us from drawing any causal conclusions about the relationship between meat attachment and meat consumption. Although the study highlights a strong association, future longitudinal studies are required to establish whether changes in meat attachment lead to subsequent reductions in meat intake. Second, the data rely heavily on self-reported measures, which are subject to recall bias and social desirability bias. Participants may have underreported their meat consumption or overestimated their desire to reduce it, especially given the increasing public discourse surrounding plant-based diets and environmental sustainability. Self-reported data on dietary habits should be complemented with objective measures, such as food diaries or biomarkers of meat consumption, to enhance the accuracy of the findings. Third, the study was conducted exclusively among primary care patients in the Geneva area. While this offers valuable insights into a specific population, the results may not be generalizable to other regions or cultures with different dietary habits and attitudes toward meat. Geneva, a relatively affluent and urban area, might not reflect the perspectives of rural populations or those from lower socioeconomic backgrounds, where meat may play a more central role in daily nutrition. Further research in diverse settings is necessary to validate the MAQf-4 in a broader range of populations. Finally, the sample size, though adequate for a preliminary analysis, also presents limitations in terms of subgroup comparisons. For example, while we explored the association between meat attachment and consumption across gender and age, a larger sample would be necessary to detect more nuanced differences or interactions between demographic factors. Future studies with more participants could help unravel whether specific groups, such as younger individuals or those with particular cultural backgrounds, exhibit unique patterns of meat attachment.

## Conclusions

The MAQf-4 is a concise tool that effectively measures attachment to meat, offering a practical alternative to the original 16-item MAQ. This cross-sectional study shows that higher MAQf-4 scores are strongly associated with increased meat consumption among primary care patients in the Geneva area. These findings suggest that psychological attachment to meat may play an important role in shaping meat consumption patterns, with potential implications for public health and environmental sustainability.

The MAQf-4 could potentially be integrated into clinical settings to help healthcare providers identify individuals with strong emotional or cognitive ties to meat and support more personalized dietary counseling. In research settings, the MAQf-4 may serve as a useful instrument for assessing meat attachment and examining interventions aimed at promoting more sustainable and health-conscious eating habits. Overall, the MAQf-4 represents a practical tool for clinical and research contexts to better understand meat attachment.
